# Editorial: New Horizons in Food Science via Agricultural Immunity

**DOI:** 10.3389/fnut.2020.00019

**Published:** 2020-02-28

**Authors:** Tomonori Nochi, Corné M. J. Pieterse, Willem Van Eden

**Affiliations:** ^1^Graduate School of Agricultural Science, Tohoku University, Sendai, Japan; ^2^Department of Biology, Utrecht University, Utrecht, Netherlands; ^3^Department of Veterinary Medicine, Utrecht University, Utrecht, Netherlands

**Keywords:** agricultural immunology, innate immunity, plants, animals, fishes

The excessive use of drugs such as antibiotics and pesticides in the fields of livestock and plant production has a significant impact on not only spreading the drugs in production sites of agriculture but also threatening human health. In particular, the use of antibiotics in agriculture has been considered to be a major factor in producing antibiotics-resistant bacteria that often infect humans. Although the evidence was firstly confirmed in the United Kingdom as “Swann Report” in 1969 (1), the issue had not yet been debated in depth for more than a quarter century. However, in recent years, the World Health Organization (WHO) has warned that infectious diseases caused by antibiotics-resistant bacteria will devastate us human beings. In addition, WHO recently adopted a proposal entitled “Global Action Plan on Antimicrobial Resistance” to avoid the crisis of such infectious diseases on a global scale (2). It should be noted that the number of people who die due to the infectious diseases caused by the antibiotics-resistant bacteria in 2050 has been estimated to be over 10 million, which exceeds the number of cancer deaths if no action is taken immediately (3).

Agricultural immunology is a novel research field that focuses on animal and plant immunology. Recent progress in agricultural immunological research has demonstrated that the innate immune system in animals and plants against pathogens that cause agricultural infection occasionally has common features regardless of the presence or absence of acquired immunity. In 2016 in Leiden, the Netherlands, a workshop entitled “Innate Immunity of Crops, Livestock and Fish: The Dawn of Agricultural Immunology” was organized by Leiden University Lorentz Center. Through the workshop, the current knowledge and recent findings regarding agricultural immunology in animals and plants were discussed interdisciplinary by participants who attended the workshop from the Netherlands, Japan and the US to create a knowledge platform necessary for future drug-independent agricultural production systems (4). Through the workshop, several possible approaches to reduce the amount of antibiotics and pesticides from the production sites of agriculture were considered. In addition, an idea of creating a Research Topic for Frontiers in Nutritional Immunology was proposed to precisely appreciate the importance of agricultural immunology.

In this Research Topic entitled “New Horizons in Food Science via Agricultural Immunity,” 11 articles including 7 review articles, 3 original research papers, and 1 brief research report were published. Current knowledge of the innate immune function in the livestock intestine where multiple pathogens continuously infect was precisely summarized in a review article based on the results obtained using *in vitro* bovine intestinal epithelial cells (Villena et al.). Also, in one other review article an approach to stimulate immune functions in not only livestock but also chickens was comparatively discussed, proposing an effective vaccine strategy for preventing infectious diseases (Nochi et al.). A recent advance of metagenomics approach using a next generation sequencer has allowed identifying numerous micro-organisms cohabiting in animals and plants. Since most of the micro-organisms identified by the new technology are anaerobic (thus cannot be cultured under general aerobic conditions), they had been a black box in bacteriology for a long time. Three review articles focused on understanding the role of such beneficial microorganisms that affect the innate immune function in the host (Ikeda-Ohtsubo et al.; Villena, Kitazawa et al.; Brugman et al.). A possible approach proposed by the review articles is to utilize the beneficial microorganisms as probiotics for animals and plants. In this regard, the two original research papers gained deeper insights into the understanding the function and characterization of such probiotic candidates (i.e., *Lactobacillus reuteri* and *Lactobacillus delbrueckii*) (Giri et al.; Kanmani et al.). Another important information obtained in this Research Topic were the presence of immune deviant phenotype observed in some cattle regardless of the vaccination status (Lutterberg et al.), the role of milk-derived extracellular vesicles including microRNA in the regulation of cell functions in offspring (van Herwijnen et al.), the effect of maillard reaction products and advanced glycation end-products on immunomodulation in animals (Teodorowicz et al.), and the function of bovine immunoglobulin in immune function, allergy and infection (Ulfman et al.). In conclusion, the topic provided multiple possibilities to strengthen the innate immune activity of animals and plants to reduce the use of antibiotics and pesticides in agriculture. Based on the knowledge updated in this topic, further additional studies should be carried out especially at the production sites of agriculture to establish the drug-independent food safety system.

## Author Contributions

All authors listed have made a substantial, direct and intellectual contribution to the work, and approved it for publication.

### Conflict of Interest

The authors declare that the research was conducted in the absence of any commercial or financial relationships that could be construed as a potential conflict of interest.
